# Long-Term Follow-Up of the Telemonitoring Weight-Reduction Program “Active Body Control” 

**DOI:** 10.1155/2016/3798729

**Published:** 2016-03-21

**Authors:** Gabriele Stumm, Alexandra Blaik, Siegfried Kropf, Sabine Westphal, Tanja Katrin Hantke, Claus Luley

**Affiliations:** ^1^4sigma GmbH, Bajuwarenring 19, 82041 Oberhaching, Germany; ^2^Institute of Clinical Chemistry and Pathobiochemistry, Otto-von-Guericke University, Leipziger Straße 44, 39120 Magdeburg, Germany; ^3^Institute of Biometry and Medical Informatics, Otto-von-Guericke University, Leipziger Straße 44, 39120 Magdeburg, Germany; ^4^Municipal Hospital Dessau, Auenweg 38, 06847 Dessau-Roßlau, Germany; ^5^Die Schwenninger Krankenkasse, Spittelstraße 50, 78056 Villingen-Schwenningen, Germany

## Abstract

The Active Body Control (ABC) weight-reduction program is based on telemonitoring of physical activity and nutrition together with telecoaching by weekly counseling letters sent by post or by e-mail. The study presented here reports the results of a 1-year follow-up of 49 patients with the metabolic syndrome who had lost weight with the aid of the ABC program in the preceding year. The weight regain after the second year in patients not receiving any further care (“ABC discontinued” group; *n* = 24) and the potential benefit of continuing with the ABC program with monthly counseling letters (“ABC continued” group; *n* = 25) were investigated. The relative weight changes after the first year had been, respectively, −13.4% and −11.4% in the “ABC discontinued” and “ABC continued” groups, and after the second year they decreased by, respectively, 4.4 and 2.8%. However, this difference in weight regains between the two groups was not statistically significant. It is concluded that three-quarters of the weight loss after 1 year is maintained after the second year. The decision whether to continue with the ABC program after 1 year should be made individually.

## 1. Introduction

Epidemiological data show that the prevalence of obesity is increasing steadily despite the implementation of various weight-reduction programs. Either the measures used at present are insufficiently effective or the successful interventions are resources-intensive and not easily translated into routine primary care. In addition, long-term maintenance of the weight loss is a problem. New more effective and economic alternatives are therefore urgently needed.

Mobile technology and web-based approaches to lifestyle improvements are constantly being developed and can improve short-term weight losses. However, the published data differ markedly in the technologies used, patient populations studied, and study durations. Recent reviews agree that further research is necessary, especially with the aim of identifying success factors for long-term weight loss maintenance [1–3].

The Active Body Control (ABC) program is a lifestyle intervention that combines telemonitoring of daily physical activity and calorie intake with weekly individual feedback by experienced coaches over 6–12 months. This program was first shown to be effective in obese adults [4] and in patients with type 2 diabetes mellitus [5].

Below are presented data from a continuation of a preceding intervention study in which patients with the metabolic syndrome had participated for 12 months. As reported previously [6], these patients had reduced their initial body weight by 11.4%. To investigate the long-term effect of the ABC program, the patients were followed up for another 12 months. The objective of this extension study was to answer two questions: first, to what extent the patients were able to maintain their weight loss, and, second, whether continuation of the telemonitoring and telecoaching in a subgroup can prevent or slow down weight regain.

## 2. Materials and Methods

### 2.1. Patients and Interventions

Patients recruited in several major companies and by the University of Magdeburg from several regions in Germany met the criteria for diagnosis of the metabolic syndrome according to the recommendations of the International Diabetes Federation [7]. The exclusion criteria were as follows: age below 30 or above 60 years and presence of diabetes mellitus, coronary heart disease, chronic heart failure, cerebrovascular disorders, or other conditions possibly also having a bearing on physical activity or body weight, such as psychiatric disorders, use of antidepressants, neuroleptic or cortisol therapy, thyroid dysfunction, active cancer or other severe diseases, disabling disorders of the motor system, pregnancy, or changes in oral contraception. None of these patients had taken part in an earlier study carried out by the authors. Details of the study design have been described previously [6].

All patients attended an initial 2-hour instruction meeting including an explanation of the Magdeburg Dual Diet. This consists of conventional calorie restriction, with reduction in the calorie intake by 500 kcal/day and preference for carbohydrates with low glycemic index. The importance of daily moderate but regular physical activity was emphasized. To this end, the patients were provided with the Aipermotion 440 model from Aipermon GmbH (Munich, Germany). The accelerometers were programmed individually for each patient and calculated the daily walking distances and daily exercise-related energy expenditure in kilocalories and in addition recorded meal calories in a simplified form. The actual balance between total calorie intake and calories used up by the basal metabolic rate plus physical activity could be checked by the carrier at any time. The validity of these measurements has been demonstrated satisfactorily by comparison with spiroergometry in various patient groups with heart failure [8] and by comparison with the 6-minute-walk test [9]. These data and self-reported daily body weights were transmitted once a week to a server in Magdeburg University Hospital by means of a USB connection to a home computer with automatic extraction of new data. During the first 12 months of the intervention, the ABC carers (physicians and ecotrophologists qualified in nutritional medicine) generated weekly individual report letters using the ABC platform (further details in [6]).

**Figure 1:**
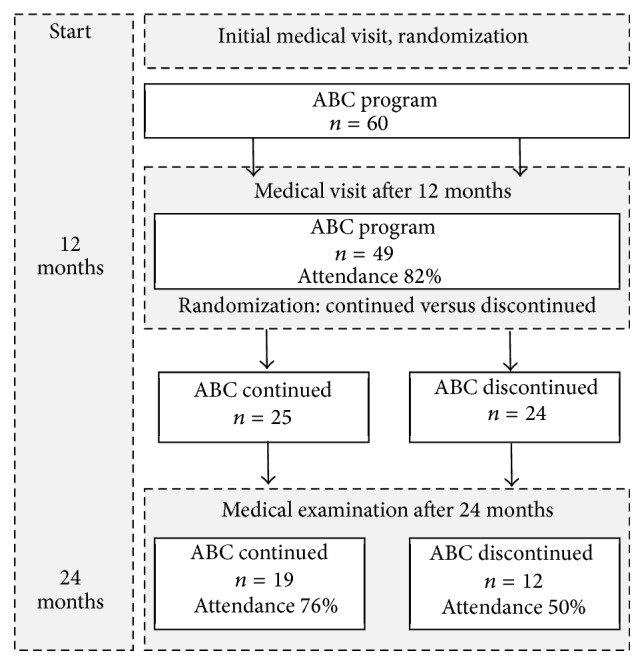
Study design: patients who completed the one-year program [6] were randomized into two groups. The “ABC continued” group continued telemonitoring and received telecoaching for a second year and was counseled by monthly letters. The “ABC discontinued” group was not contacted for another 12 months until the medical examination after 24 months.

The study was then continued for a second year. All 49 patients who had finished the first year were randomized to one of two groups. In the first group the counseling was continued, but with lower intensity than during the first year (“ABC continued” group, *n* = 25). During the first year all patients had received counseling letters every week. This frequency was reduced during the second year to one letter per month. In the second group both telemonitoring and feedback by counseling letters were completely discontinued (“ABC discontinued” group, *n* = 24). At the end of the second year both groups were invited for a final medical examination. The design of the study is shown in Figure 1.

The study had been approved by the Ethics Committee of the School of Medicine and all subjects had given their written informed consent.

### 2.2. Statistical Analysis

The patients were randomized to the “ABC continued” and “ABC discontinued” groups by lot.

Owing to the dropout rate before the final examination, the analyses can only claim to be exploratory. Nevertheless, methods appropriate for analyses with incomplete data were used, to avoid bias as far as possible.

Analyses for the outcome parameters including relative weight loss (in percentage of the weight at baseline) are based on a mixed model for repeated measurements (software SAS, PROC MIXED). Each outcome parameter was analysed separately. The model is based on differences of the outcome parameters from their corresponding baseline values as dependent variables. The baseline values are included as covariables. The study subjects are modeled as random factors (random intercept). All estimates given in the text are based on this model. Differences over time are constructed in such direction that positive values indicate an increase in the parameter in question and negative values indicate a decrease. The analysis is based on the intention-to-treat principle. Missing values due to loss to follow-up are not imputed explicitly in this approach, but all measurements available at the earlier visit are included in the model, so that missing values are implicitly taken into account.

The estimates presented here are mean changes in the outcome variable from baseline to month 12 per group, mean changes in the outcome variable from baseline to month 24 per group, and mean changes in the outcome variable from month 12 to month 24 per group, all adjusted to a common average baseline value over all treatment groups and completed by 95% confidence intervals and unadjusted *p* values for the test against zero.

## 3. Results and Discussion

### 3.1. Results

Patients who completed the first year were randomized to the “ABC continued” and “ABC discontinued” study arms.  Table 1 shows the demography of the patients who took part in the second year extension. The results of the randomization to the two groups turned out not to be quite optimal, since the mean body weight was higher in the “ABC continued” group than in the “ABC discontinued” group. However, the difference was not statistically significant in the nonparametric Mann-Whitney-Wilcoxon test, with *p* = 0.136.

Table 2 shows relative and absolute weight changes and BMI values after 12 and 24 months in the two groups, with corresponding levels of statistical significance of the differences. The weight regain values during the second year are given in columns difference 24 − 12 and p3^*∗*^. It is apparent that significant weight regains had occurred, whether or not the ABC telemonitoring was continued. The regain was higher in the “ABC discontinued” group, with plus 4.4% points relative to the initial weight in comparison with 2.8% points in the “ABC continued” group. However, this difference in relative weight regain between the two groups was not statistically significant, as maybe seen from the column p4 contd. versus discontd.

### 3.2. Discussion

Various interventions aimed at weight reduction have been demonstrated to be effective in short-term studies, but long-term interventions and weight-loss maintenance have been addressed less frequently (reviews in [10, 11]). Existing data suggest that weight regain is a frequent problem and begins 12–24 months after the initiation of the weight reduction. To investigate the long-term effect of intervention by the ABC program, patients who had taken part in this program for 1 year were followed up for another 12 months. The objective was to answer two questions: first, were the patients able to maintain their reduced weight, and, secondly, can continuation of telemonitoring and telecoaching in a subgroup prevent or slow down weight regain?

The answer to the first question is unequivocal: the patients did regain weight during the second year. To obtain a relative assessment of the weight regain in our study we researched current literature for long-term results. It turned out that publications reporting long-term results are rare, in particular, ones with similar types of intervention and comparable time-points of the measurements. We identified seven long-term studies in which weight had been reduced by lifestyle interventions. Studies using formula diets or bariatric surgery were excluded.

The lowest weight regain, by 0.8% points relative to the original maximum weight loss, was observed in Volger's “brief lifestyle counseling” group [12]. 131 participants received monthly coaching during the second year. The relative weight change was only −3.5% after 12 months and −2.7% after 24 months, which is below the widely acknowledged minimum weight loss target of 5% required for obese patients [13, 14].

**Figure 2:**
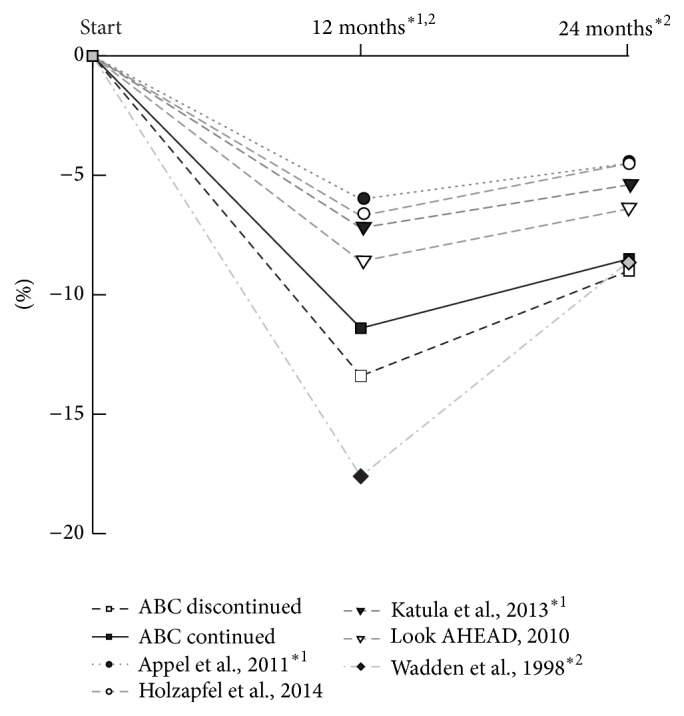
Comparison of weight-reduction data after 12 and 24 months in seven long-term studies including the present study. Only those studies are shown in which a minimum weight loss of 5% of the initial body weight was achieved after 12 months.  ^*∗*1^Appel et al.  and Katula et al.: weight measures at 6 and 24 months.  ^*∗*2^Wadden et al.: weight measures at 48 and 100 weeks.

The other six trials analysed reached the minimum weight reduction of 5% relative to the baseline weight after 12 months [15–20], but only three of them, and also the ABC program, achieved this >5% target after 24 months ([18–20] Figure 2). Appel et al. used a web-based education approach in addition to group meetings in 139 obese patients and achieved a weight change of −6% in the first 6 months. After the second year the patients' weight had risen by 1.5% points despite monthly coaching [15] and had therefore missed the minimum target of 5% weight reduction. Similar results were reported for two other interventions. A weight change of −7.2% of the baseline body weight was observed in 772 participants in the commercial weight watcher program [16] after 12 months. Left unattended for the second follow-up year, they regained 2.7% points, which reduced the weight change to −4.5% of the initial body weight after 24 months. This result might even be overestimated, because it is based on only 26% of the initial participants who attended the final examination. A rather high weight regain was observed by Jakicic et al. [17], who used an intervention based on eating behavior and exercise in 191 women. After 12 months their body weight had changed by −8.9% but rose again by 3.5% after 24 months, resulting in a long-term weight change of −4.6%.

The goal of maintaining at least a 5% weight loss after two years was reached in the HELP-PD and the Look AHEAD trials. The Healthy Living Partnerships to Prevent Diabetes Study (HELP-PD) [18] used community health workers with well controlled type 2 diabetes and history of healthy eating and physical activity for peer coaching 301 type 2 diabetes patients. These subjects achieved a weight change of −7.2% after 12 months. Although their weight rose again by 1.8% points despite continued monthly coaching, the final weight change after 2 years was −5.4%. Still greater weight loss was obtained in the large-scale Look AHEAD trial [19], in which 2570 participants were assigned to an intensive lifestyle intervention with a calorie reduction diet including low fat and elevated protein intake as well as at least 175 minutes of physical activity per week. After 12 months the intervention group showed a weight change of −8.6% relative to the baseline. However, monthly face-to-face meetings and additional contacts once a month by telephone or by e-mail could not prevent weight regain by 2.6% points, which resulted in a 2-year weight change of −6.3%. It should be mentioned that the Look AHEAD Research Study Group has now published additional data reporting weight gains after 4 and 8 years [20]. The weight gain continued for up to 4 years, but then the participants appeared to stabilize. After 8 years a mean weight change of −4.7% was still present, and the intensive lifestyle intervention group produced clinically meaningful weight loss (≥5%) in year 8 in 50% of the patients with type 2 diabetes.

The highest initial weight loss was reported by Wadden et al. [21], who applied an intensive dietary and fitness training program in 99 women with a mean age of 42 years. An impressive −17.6% weight change loss was achieved after 48 weeks. However, the long-term weight progress was disappointing. The participants regained more than half of the initial weight loss, by plus 9.0% points, in the second year in the absence of continued treatment. The final weight change after 2 years was therefore −8.6%, which is very close to the 2-year weight changes in our two study arms.

Concerning weight regain, it may be concluded that weight regains in the second year are observed in all weight-reduction programs, including the ABC program. In order to maintain a medically relevant weight loss also after the second year, it is therefore very important that the weight reduction in the first year should be large enough and that the subsequent weight regain is moderate. From this point of view, the overall results of ABC program are quite satisfactory.

Our second question was whether continued ABC coaching in the second year, though less frequent, could still have a positive influence on the regain in a subgroup of our patients. The data of our study do not allow a clear answer. The difference in weight regain between the “ABC continued” and “ABC discontinued” group indicates a lower regain in the “ABC continued” group, but this difference was not significant, perhaps because of the small number of patients and the high dropout rate in the “ABC discontinued” arm. A more detailed analysis showed, however, 4 patients in the discontinued arm with a regain of more than half of the initial weight loss. In contrast, no weight regains of this magnitude were observed in any of the patients of the “ABC continued” arm. This tendency toward smaller weight regains with continued counseling has been mentioned repeatedly in the literature described above. However, until a clear benefit of prolonged coaching has been confirmed by studies with greater numbers of patients we recommend to make the decision to stop or continue coaching in each case on the basis of the individual situation, such as the patient's remaining excess weight, health status, willingness to cooperate, and social aspects.

A major difference between the ABC program and the other weight-reduction programs in Figure 2 is that the ABC program uses a telemedical approach, while all other studies relied mainly on repeated counseling sessions. There was only one face-to-face meeting in the course of the 24 months of the ABC program, as compared with, respectively, 28, 33, 44, and 66 such meetings in the studies of Wadden et al., Appel et al., and Katula et al. and in the Look AHEAD trial. Despite this short personal encounter, the weight reduction achieved in the ABC program was second best after 12 months and best after 24 months. We believe that this satisfying efficacy of the telemedical approach is due to the fact that it moves the obesity treatment from the counselor's office into the patient's daily life. The telemonitoring approach enforces the patient's continuous self-control which is boosted by the regular motivation letters from carers who are closely connected with the patients and who respond individually. It remains to be investigated whether additional face-to-face contacts would further enhance the efficacy of the ABC program.

The telemedical character of our program yields yet another advantage worth mentioning: the time expenditure is much smaller. The single instruction meeting at the start of the program takes 2 hours for both carers and patients. Daily physical activity is measured automatically, and documentation of calorie intake is simple and takes the patient only a few minutes. The preparation of the regular individual report letter takes the carer 5 minutes.

The small time expenditure by the patients might contribute to their compliance with the program, which during the second year was relatively high (75%) and well comparable with the compliance figures reported in most of the other studies discussed here (77–94%). In general, however, the rates of compliance in weight-reduction studies can be very variable, ranging from 20 to 90% [22]. As an example of a commercial program, in the weight-watcher intervention described by Holzapfel et al. [16] it was only possible to bring back 26% of the participants for the 24-month visit.

Finally, the small time requirement of the ABC program results in low costs. Although the studies discussed did not specify the expense of their programs, Tsai et al. investigated the costs for a number of conservative obesity treatments over 2 years: the costs per kg-year were $219–$437 per kg in usual care with quarterly counseling sessions and $292 for an “enhanced brief lifestyle treatment” with monthly meetings [23]. Because of the greater weight loss, the corresponding figure for the ABC program is much better, $47 per kg-year.

## 4. Conclusion

The ABC telemonitoring weight-reduction program yields a weight loss that, in comparison with other programs, remains favorable even after two years. The decision to continue the program for a second year should be made individually.

## Figures and Tables

**Table 1:** Demography of participants who were followed up for a second year. The mean weight in the “ABC discontinued” group was somewhat lower than in the “ABC continued” group, but the difference did not reach statistical significance (*p* = 0.136). The figures given are means and standard deviations.

	ABC continued (*n* = 25, 36% female)	ABC discontinued (*n* = 24, 29% female)	*p*: continued versus discontinued
Age (years)	50 ± 8	52 ± 7	n.s.
Weight (kg)	95.5 ± 15	88.1 ± 13.5	n.s.
Height (cm)	1.76 ± 0.1	1.76 ± 0.1	n.s.
BMI	30.9 ± 5.1	28.5 ± 3.9	n.s.

**Table 2:** Relative and absolute weight changes and BMI values changes at 12 and 24 months in the group which continued to receive ABC telecoaching (“ABC contd.”) after 12 months and in the group which did not receive further telecoaching (“ABC discontd.”). The figures given are means and standard deviations.

	Study groups	12 months	p1 0 versus 12	24 months	p2 0 versus 24	Difference 24 − 12	p3 24 versus 12	p4 contd. versus discontd.
Weight change (%)	ABC contd.	**−11.4**	<0.0001	**−8.5**	<0.0001	**2.8**	<0.0001	n.s.
		*(−13.7; −9.1)*		*(−11.0; −6.1)*		*(−4.3; −1.4)*		
	ABC discontd.	**−13.4**	<0.0001	**−9.0**	<0.0001	**4.4**	<0.0001	
		*(−15.8; −11.1)*		*(−11.6; −6.4)*		(6.1; −2.8)		

Weight (kg)	ABC contd.	**−11.8**	<0.0001	**−8.9**	<0.0001	**2.9**	0.0001	n.s.
		*(−14.3; −9.4)*		*(−11.5; −6.4)*		*(−1.5; −4.4)*		
	ABC discontd.	**−14.6**	<0.0001	**−9.8**	<0.0001	**4.7**	<0.0001	
		*(−17.03; −12.1)*		*(−12.6; −7.1)*		*(−3.0; −6.5)*		

BMI (kg/m^2^)	ABC contd.	**−4.1**	<0.0001	**−3.1**	<0.0001	**1.0**	<0.0001	n.s.
		*(−4.9; −3.3)*		*(−3.9; −2.3)*		*(−0.5; −1.5)*		
	ABC discontd.	**−4.8**	<0.0001	**−3.3**	<0.0001	**1.5**	0.0005	
		*(−5.6; −4.0)*		*(−4.2; −2.4)*		*(−0.9; −2.0)*		

p1 0-12: 0 versus 12 months (changes are losses over 12 months).

p2 0-24: 0 versus 24 months (changes over 24 months).

p3 24-12: 24 versus 12 months (smaller changes, weight regains in the second year).

p4 contd. versus discontd.: difference 24 − 12 months for continued versus discontinued intervention.
